# Graft cell expansion from hiPSC-RPE strip after transplantation in primate eyes with or without RPE damage

**DOI:** 10.1038/s41598-024-60895-w

**Published:** 2024-05-02

**Authors:** Keisuke Kajita, Mitsuhiro Nishida, Yasuo Kurimoto, Satoshi Yokota, Sunao Sugita, Toshika Semba, Satoshi Shirae, Naoko Hayashi, Atsuta Ozaki, Yoko Miura, Akiko Maeda, Yoshinori Mitamura, Masayo Takahashi, Michiko Mandai

**Affiliations:** 1 Kobe City Eye Hospital, 2-1-8, Minatojima-minamimachi, Chuo-ku, Kobe, Hyogo 650-0047 Japan; 2 Department of Ophthalmology, Institute of Biomedical Sciences, Graduate School, Tokushima University, Tokushima, Japan; 3 Department of Ophthalmology, Mie University Graduate School of Medicine, Tsu, 514-8507 Japan; 4 Laboratory for Retinal Regeneration, RIKEN Center for Biosystems Dynamics Research, 2-2-3, Minatojima-minamimachi, Chuo-ku, Kobe, Hyogo 650-0047 Japan; 5 Vision Care Inc. Kobe Eye Center 5F, 2-1-8 Minatojima-minamimachi, Chuo-ku, Kobe, 650-0047 Japan; 6 Institute of Biomedical Optics, University of Lübeck, Peter-Monnik-Weg 4, 23562 Lübeck, Germany; 7 Department of Ophthalmology, University of Lübeck, Ratzeburger Allee 160, 23562 Lübeck, Germany

**Keywords:** Regeneration, Preclinical research, Translational research, Retinal diseases

## Abstract

Clinical studies using suspensions or sheets of human pluripotent cell-derived retinal pigment epithelial cells (hiPSC-RPE) have been conducted globally for diseases such as age-related macular degeneration. Despite being minimally invasive, cell suspension transplantation faces challenges in targeted cell delivery and frequent cell leakage. Conversely, although the RPE sheet ensures targeted delivery with correct cell polarity, it requires invasive surgery, and graft preparation is time-consuming. We previously reported hiPSC-RPE strips as a form of quick cell aggregate that allows for reliable cell delivery to the target area with minimal invasiveness. In this study, we used a microsecond pulse laser to create a local RPE ablation model in cynomolgus monkey eyes. The hiPSC-RPE strips were transplanted into the RPE-ablated and intact sites. The hiPSC-RPE strip stably survived in all transplanted monkey eyes. The expansion area of the RPE from the engrafted strip was larger at the RPE injury site than at the intact site with no tumorigenic growth. Histological observation showed a monolayer expansion of the transplanted RPE cells with the expression of MERTK apically and collagen type 4 basally. The hiPSC-RPE strip is considered a beneficial transplantation option for RPE cell therapy.

## Introduction

Clinical studies have been conducted globally on the transplantation of pluripotent stem cell-derived retinal pigment epithelium (RPE) as a promising therapeutic option for the treatment of diseases with RPE functional impairment, including age-related macular degeneration^1–8^. Two major approaches have been presented as methods of transplantation: cell suspension and RPE sheet transplantation. Cell suspension injection requires minimal surgical invasion; however, cell leakage is inevitable, resulting in a non-conclusive number of delivered cells and frequent formation of the epiretinal membrane. Moreover, targeting some specific areas to deliver a substantial number of cells using this approach is challenging^5,8^. Conversely, RPE sheet transplantation allows for the reliable delivery of cells to the target site with the correct cell polarity. However, preparing a sheet requires weeks of cell culture, an expensive and laborious process. Transplantation of a large sheet requires invasive surgery with a large incision in the sclera and retina, which may increase the risk of surgical complications, including hemorrhage and retinal detachment^4–9^.

Previously, these concerns were addressed by using a human pluripotent cell-derived retinal pigment epithelial cell (hiPSC-RPE) strip where we can form strip-like aggregates by incubating them in narrow grooves for 2 days, and RPE cells can migrate out from the strip placed in a dish, expanding while retaining a characteristic cobblestone-like appearance with the secretion of vascular endothelial growth factor and pigment epithelium-derived factor^10^. Furthermore, we observed that after transplantation in immune-deficient nude rats, RPEs expanded from the hiPSC-RPE strip as a monolayered RPE that was positive for RPE markers, RPE65 and MERTK, and contacted host photoreceptor outer segments. The expression of apical marker Ezurin and basal marker type IV collagen demonstrated correct polarity in both these monolayered RPE and the top layers of multilayered RPE strip clusters.

In clinical cases with RPE impairment, RPE cells are literally lost or degenerated in some cases, whereas they may be dysfunctional but morphologically present and alive in other cases. Considering the clinical application of hiPSC-RPE-strips, understanding how the hiPSC-RPE cells migrate and engraft in these different environments in vivo is essential. Therefore, in this study, we ablated RPE cells using microsecond pulse laser photocoagulation and investigated how hiPSC-RPE cells are engrafted in the area in the presence or absence of damaged RPE cells.

## Results

### Optimization of RPE ablation by microsecond pulse laser treatment

**Figure 1:**
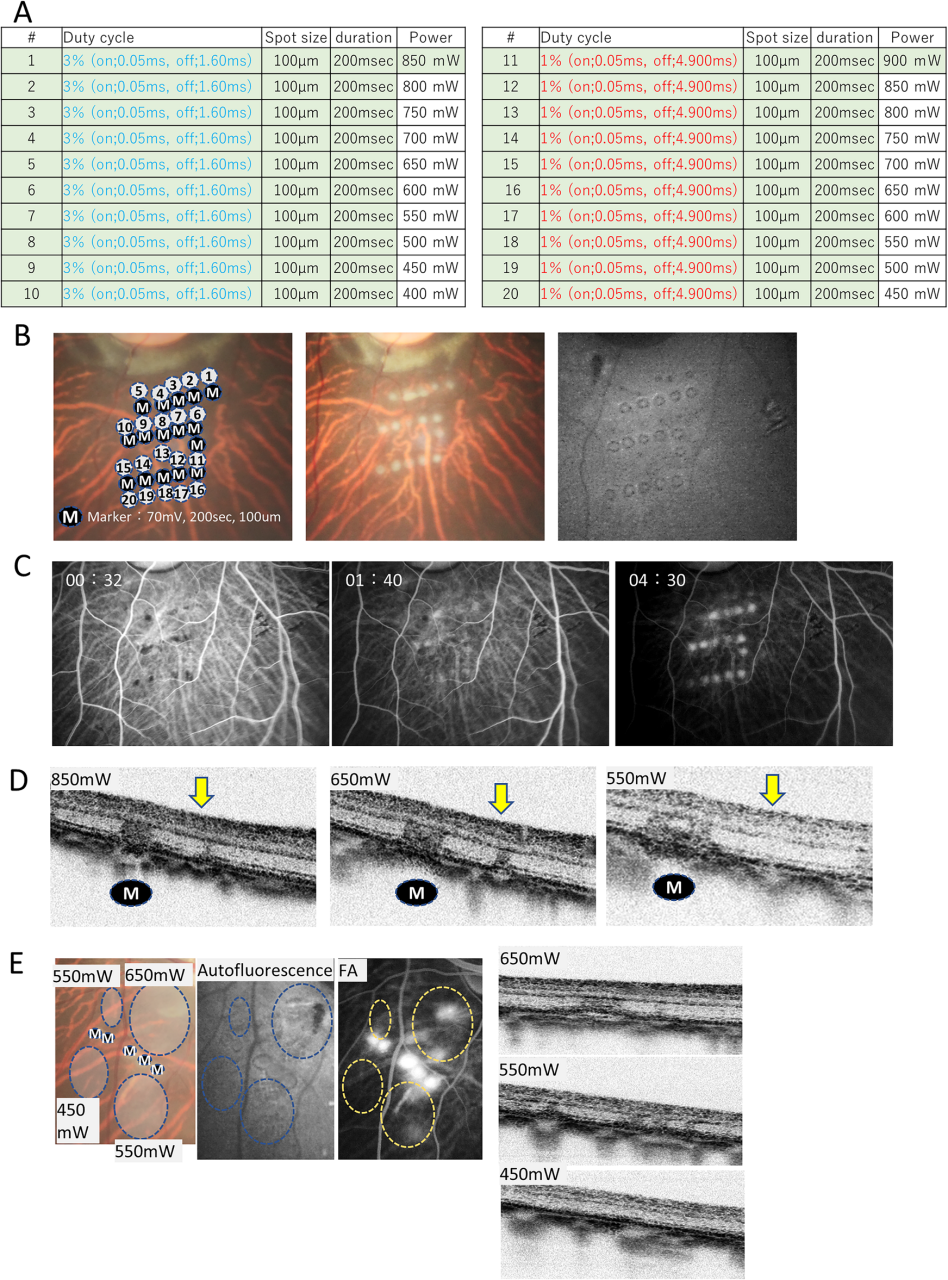
Optimization of microsecond pulse laser conditions. (**A**) Table summarizing the tested conditions of the microsecond pulse laser treatment. (**B**) Retinal fundus photographs of a monkey after the irradiation of the microsecond pulse laser and autofluorescence photographs. The ‘M’ mark indicates the visible laser coagulation as a marker. The laser was irradiated at the numbered positions with the conditions listed in the table in Fig. A. Coagulated spots at numbers 1–7 are identified in the autofluorescence image. (**C**) Retinal fluorescence angiography (FA) photographs after the irradiation of the microsecond pulse laser. In the 4:30 image, coagulated spots present mild hyperfluorescence at coagulation spots 1–8. (**D**) Optical coherence tomography (OCT) image after the microsecond pulse laser. The ‘M’ mark indicates the site of laser irradiation as a marker. The yellow arrows indicate the sites where the microsecond pulse laser was irradiated. (**E**) Microsecond pulse lasers with powers of 450 mW, 550 mW, and 650 mW irradiated in a circled area. Autofluorescence photographs, fluorescent retinal angiography images, and OCT after the irradiation are shown. At 550 mW, the coagulated area showed reduced autofluorescence and a mild hyperfluorescence with FA with a mild disturbance in ONL by OCT.

We made an RPE ablation area in cynomolgus monkey eyes to mimic human RPE degeneration with cell loss based on the laser conditions used in the reported porcine model (Fig. 1)^11–13^. To achieve selective RPE ablation while avoiding photoreceptor cell damage, we placed the serial test coagulations along the marker coagulation by gradually reducing the laser power by 50 mW from 850 to 400 mW with the duty cycle set at 3%, spot size at 100 μm, and duration at 200 ms (Fig. 1A, B). Although the laser burn was not clear in the fundus photograph, a clear decrease in autofluorescence was observed at 550 mW and above (#1–8 in Fig. 1B). Using fluorescence angiography (FA), dye-leakage was observed at 500–550 mW or higher (Fig. 1C). Optical coherence tomography (OCT) images showed hyperreflectivity at the marker coagulation spots, but only a very mild disturbance was observed at the ellipsoid zone line at 550 mW (Fig. 1D). With duty cycle set at 1%, we did not observe any hyperfluorescence by FA or OCT change after coagulation. To narrow down the condition, we applied 5 × 5 spot coagulation with 0-spacing with the laser powers at 450, 550, and 650 mW (Fig. 1E). At 650 mW, the coagulated area showed hypo-autofluorescence and an evident leakage with FA, accompanying some damage in the photoreceptor layer and the pigment epithelial detachment with OCT. At 550 mW, the retinal outer nuclear layer (ONL) showed increased reflex. At 450 mW, the ONL was normal; however, FA did not reveal hyperfluorescence, indicating a neglectable impact on both photoreceptors and RPEs. Based on these, the laser condition was determined by individual monkey bases between 450–550 mW by applying the test coagulation in each experiment.

### Human iPSC-RPE-strip was planned within 2 days after microsecond pulse laser treatment

**Figure 2:**
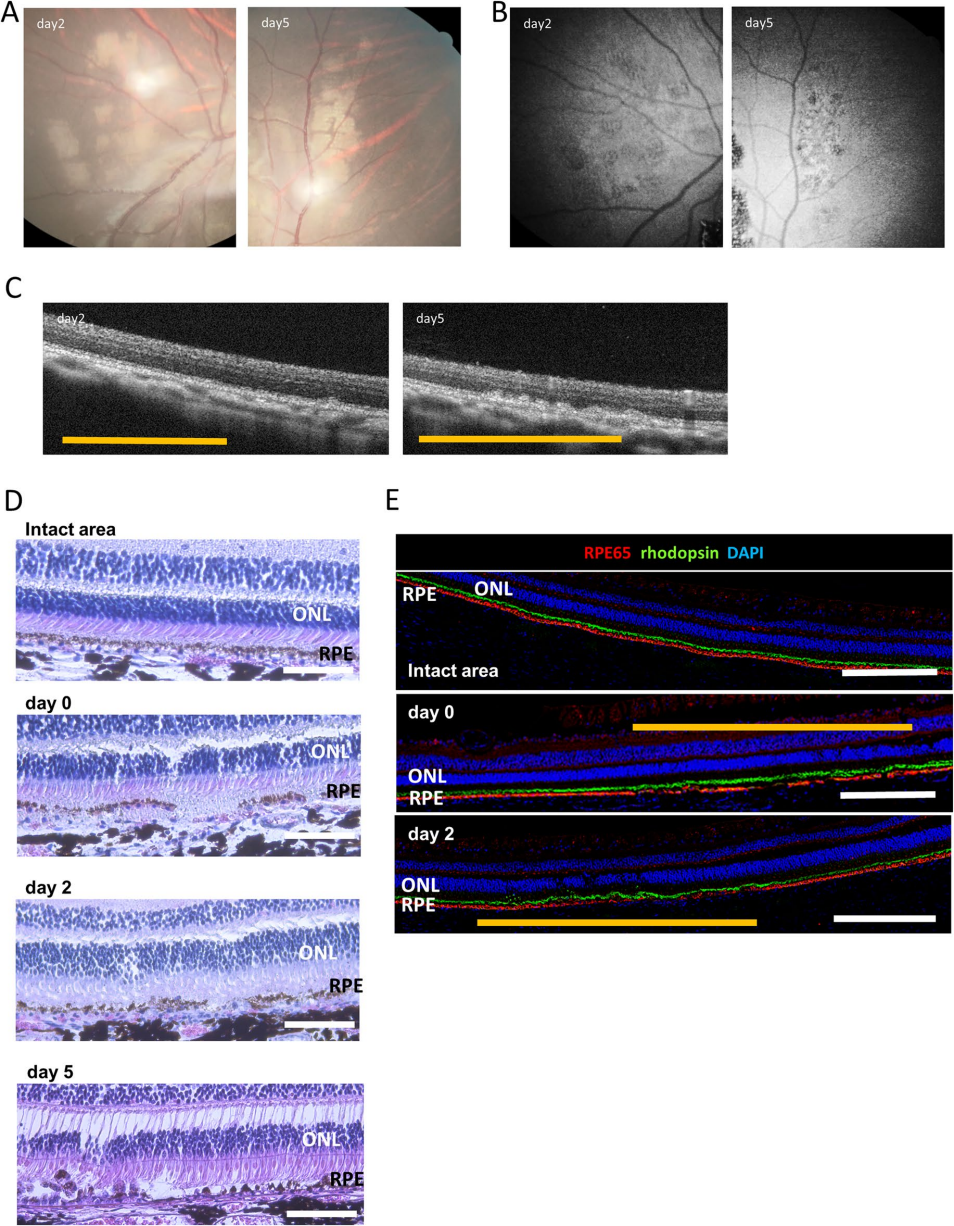
Temporal changes in the RPE layer following micropulse laser treatment. (**A**) Retinal photographs of the eyes 2 and 5 days after the application of the subthreshold micropulse laser. (B) Autofluorescence images 2 and 5 days after the subthreshold microsecond pulse laser treatment. (**C**) Optical coherence tomography (OCT) images of the coagulated site 2 and 5 days after the microsecond pulse laser treatment. The yellow line indicates the locus of laser treatment. (**D**) Hematoxylin and eosin staining of the retinal pigment epithelium (RPE) damaged site immediately after and 2 and 5 days after the microsecond pulse laser treatment. (**E**) Immunostaining of RPE65 and rhodopsin at intact and RPE-damaged areas immediately after and 2 days after the microsecond pulse laser treatment. Yellow line indicates the locus of laser treatment. *ONL* outer nuclear layer. Scale bars: (**D**) 100 μm, (**E**) 500 μm.

After determining the laser power as described above, ocular fundus photographs and OCT images were captured 2 and 5 days following the laser treatment and processed for histological observation (Fig. 2). Ocular fundus photographs taken on post-operative day 2 showed white patches at the laser site that persisted until the fifth day (Fig. 2A). The autofluorescence images exhibited reduced fluorescence at the laser-treated site after 2 days, which was more evident after 5 days. OCT analysis revealed an irregular thickening of the RPE layer after 2 days, which became more pronounced by the fifth day (Fig. 2C). No change in the thickness of the ONL was observed until the second post-operative day; however, the thickness of the ONL overlying the thickened RPE exhibited marginal attenuation on the fifth post-operative day. The laser-treated area was then studied under hematoxylin and eosin (HE) staining. (Fig. 2D). Immediately after the laser treatment, the continuity of pigmented cells at the RPE layer was irregularly disrupted, indicating a patchy loss of RPE cells. After 2 days, the proliferation and migration of surrounding RPE cells were observed. RPE alignment improved with some disruption on post-operative day 5, and some hypertrophic RPE clumps were also observed. The ONL appeared largely intact, with the presence of photoreceptor outer segments. Immunostaining of RPE65 and rhodopsin also revealed the disruption of RPE65-positive RPE cells and intact rhodopsin photoreceptor outer segments overlying the damaged RPE cells (Fig. 2E). Based on these findings, we speculated that the optimal timeframe for RPE transplantation would be 2 days following laser treatment.

### Human iPSC-RPE strip stably survived in all four monkey eyes

**Figure 3:**
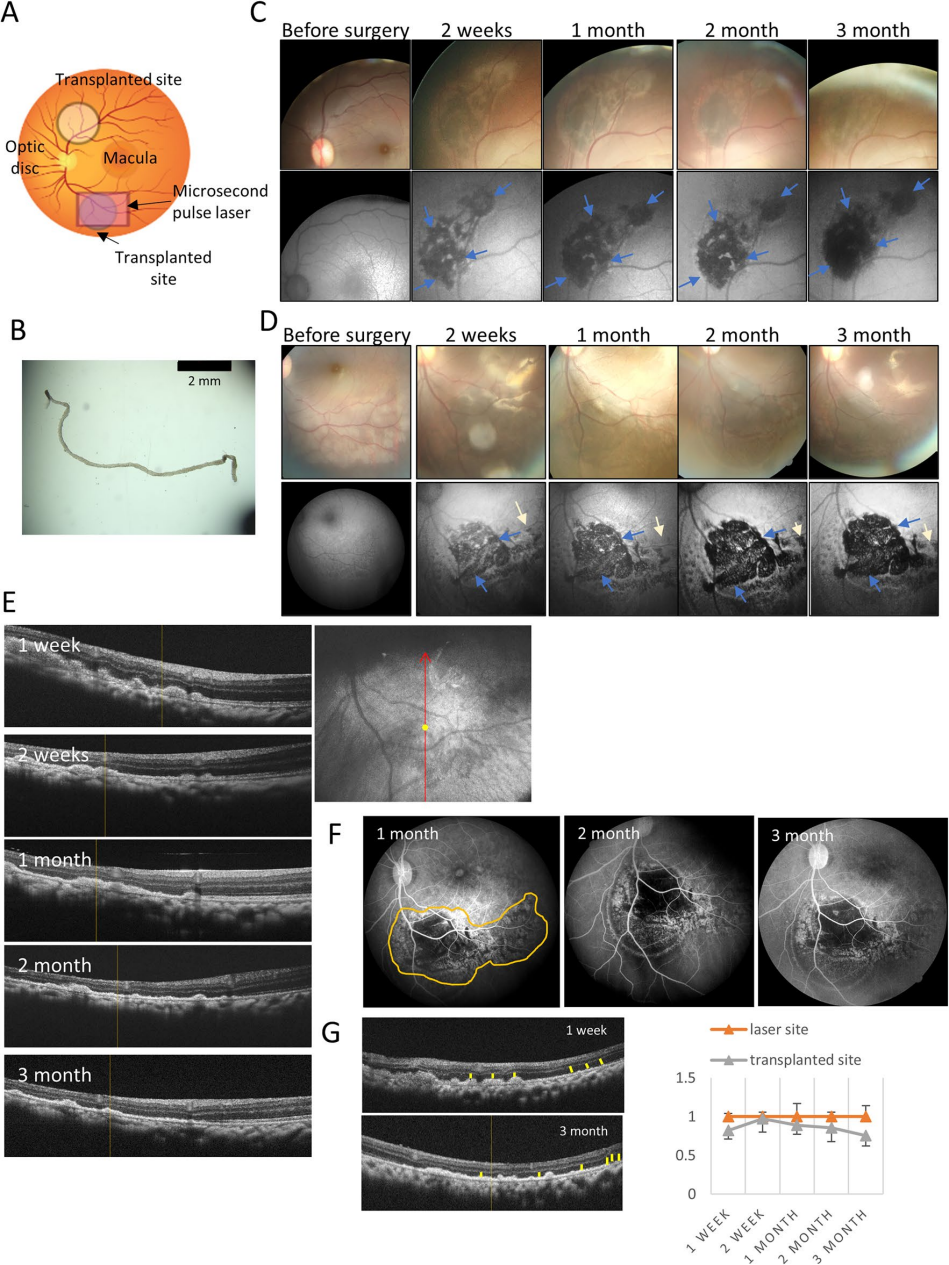
Example of temporal changes in engrafted hiPSC-RPE strips (M4). (**A**) Schematic diagram of the hiPSC-RPE strip transplantation site. RPE-damaged site created by the microsecond pulse laser treatment. hiPSC-RPE strip cells were transplanted at both the RPE-damaged and intact sites. (**B**) Image of the hiPSC-RPE strip. (**C**) Engraftment of the hiPSC-RPE strip at the intact site of the monkey eye. Expansion of hiPSC-RPE strips was observed by both color fundus and autofluorescence images. (**D**) Engraftment of hiPSC-RPE strips at the RPE-damaged site of the monkey eye. Human iPSC-RPE strips expanded from the early observation time point in both color fundus and autofluorescent images. Blue arrows indicate transplanted hiPSC-RPE, and the white arrows indicate the sites of microsecond pulse laser treatment. (**E**) Optical coherence tomography (OCT) images after iPSC-RPE strip transplantation to the RPE-damaged site. (**F**) Fluorescent retinal angiography examination after iPSC-RPE strip transplantation to the RPE-damaged site. The yellow dotted line shows the site of the microsecond pulse laser treatment and hiPSC-RPE strips expanded and block choroidal fluorescence. (**G**)Temporal change in the ONL thickness at the laser ablation site with or without transplantation. ONL thickness for both the transplant and non-transplant areas within laser injury site was measured at three locations (yellow bars) in the OCT images of the corresponding section throughout the observation period. The ONL thickness of the transplant site was shown as a mean ± sd ratio to that of the laser-only non-transplanted area. At all-time points, there was no significant difference in ONL thickness between the laser-only and implanted sites. *ONL* outer nuclear layer, *INL* inner nuclear layer.

**Figure 4:**
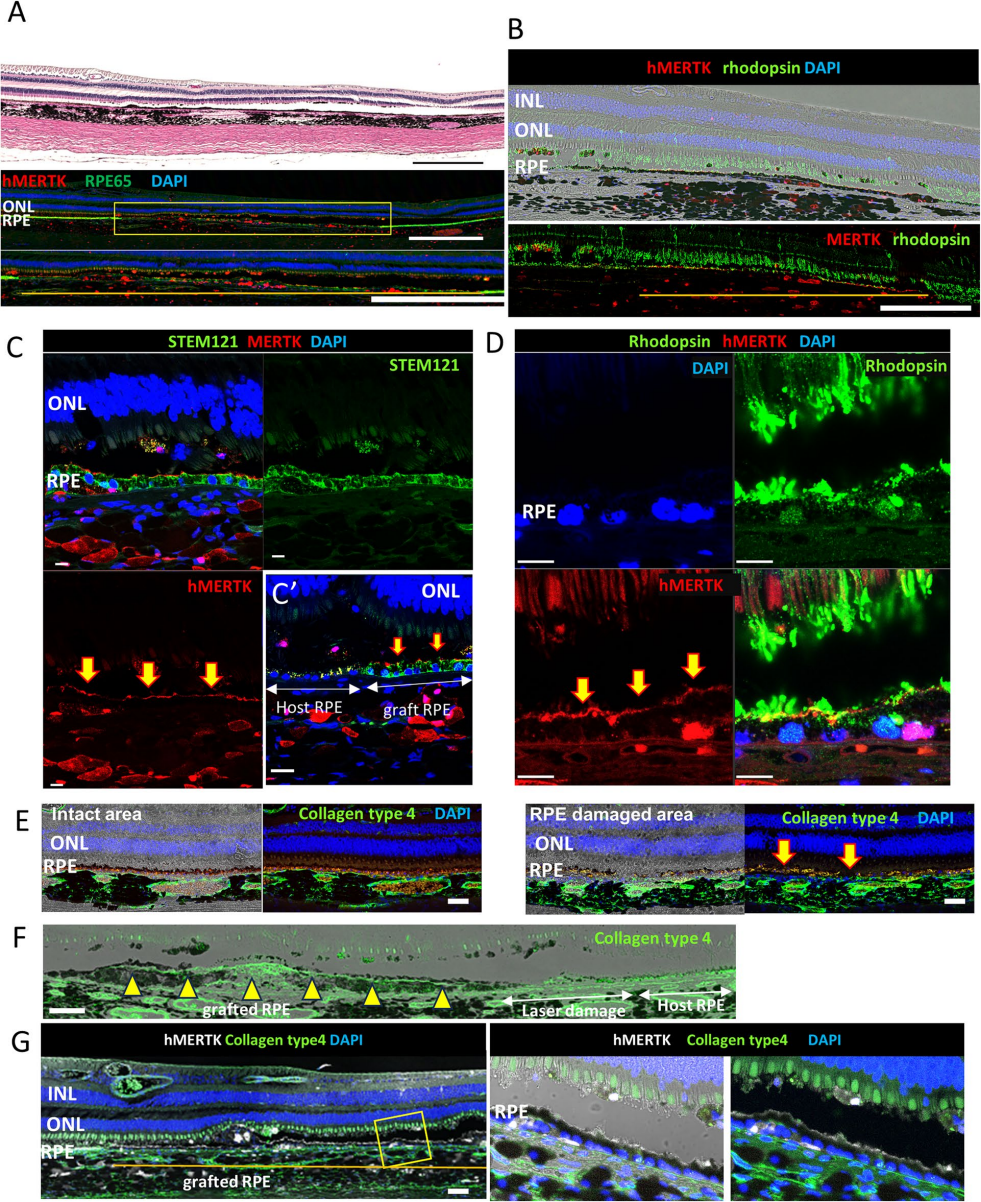
Histology of hiPSC-RPE strip transplant at an RPE-damaged site (M4). (**A**) Low magnified hematoxylin and eosin (HE) image and the immunostaining image of the neighboring section with anti-human MERTK (hMERTK) and − RPE65 antibody in the hiPSC-RPE strip transplanted area over RPE damage. Yellow line indicates MERTK positive grafted area. (**B**) Immunostaining image of hMERTK and rhodopsin. Yellow line indicates hMERTK positive grafted area. (**C**) Engrafted RPE cells are positive for STEM121 and hMERTK on the apical side (arrows) while host RPE cells at the border show only autofluorescence but not STEM121 or hMERTK positivity (C’). (**D**) hMERTK on the apical side of transplanted RPE cells (arrows) face and interact with rhodopsin-positive outer segments of host photoreceptors. (**E**) Immunohistochemistry of type IV collagen in intact and RPE-damaged sites at 2 days after laser treatment. Expression of type IV collagen is disrupted after microsecond pulse laser treatment. (**F**) Expression analysis of type IV collagen in the intact, laser-damaged, and transplant sites. Expression of type IV collagen is identified at the basement membrane of transplanted RPE. (**G**) Expression analysis of type IV collagen at the basement membrane of hMERTK-positive RPE cells. Magnified view of yellow boxed part on the right. *ONL* outer nuclear layer, *INL* inner nuclear layer. Scale bars: (**A**) 200 μm, (**B**) 100 μm, (**C**,**D**) 10 μm, (**E**) 50 μm, (**F**) 100 μm, (**G**) 50 μm.

We then conducted hiPSC-RPE strip transplantation in one eye of four macaque monkeys. Table 1 summarizes the transplanted cell-strip information and observation. In each case, hiPSC-RPE strips were transplanted in both the RPE-damaged and intact sites, except for the first monkey, which underwent the first surgery before the final optimization of the surgical protocol (Fig. 3A, B). To conduct multiple strip transplantation for clinical application, a total of four RPE strips were transplanted: two at the RPE-damaged site and two at the intact site from the second monkey. No complications were observed during the surgical procedure in any of the four eyes, and multiple hiPSC-RPE strips were safely inserted with repeated injections. One eye (M2) developed aseptic endophthalmitis within 1 week after transplantation, which was resolved with a subconjunctival steroid injection. After transplantation, the monkeys were monitored by fundus photography, autofluorescence, FA, and OCT. The representative images of the M4 examinations are shown in Fig. 3C–F (M4). The transplanted RPE strips were visualized as dark areas in autofluorescence images and the strip-like structure was identified beneath the subretinal space in OCT images obtained immediately after transplantation (Fig. S1). In the fundus photography (Fig. 3C, D), the 65RPE-damaged sites, and the pigmented area gradually increased, which was clearly visible for up to 6 months (images from 2 weeks to 3 months after surgery are presented in Fig. 3C, D). The gaps between the strips were also gradually filled over time while the RPE-damaged sites without transplantation retained almost normal autofluorescence. In the OCT images, the transplanted RPE initially looked bumpy but rapidly flattened in the first 2 weeks, whereas the ONL was retained over most of the engrafted area (Fig. 3E). On FA, the transplanted area showed a blockade of fluorescence after transplantation, whereas the RPE-damaged area with no graft showed a mild hyperfluorescence at 1 month after transplantation (Fig. 3F). No apparent fluorescence leakage, indicating graft rejection, was observed. Even in xeno-transplantation, host ONL thickness in the graft area seemed well retained away from the surgical insertion site and over the flattening graft (Fig. 3G). Hematoxylin and eosin (HE) staining revealed the presence of pigmented cells, mostly spreading as a monolayer at the RPE-damaged site (Fig. 4A). The transplanted RPE cells were positive for MERTK on the apical side, RPE65, and human surface marker STEM121 (Fig. 4A–C). This indicated the successful engraftment of the transplanted RPE cells with the correct polarity. With the present antibody, the specific pattern of apical MERTK positivity that is free of autofluorescence was only observed in the grafted human RPE cells but not in the resident monkey RPE cells (please also see Fig. S2A), and we regard this staining pattern as human MERTK (hMERTK) positivity hereafter. In addition, outer-segments were preserved over most of the grafted area, with hMERTK-positive graft RPEs interacting with rhodopsin-positive outer segments of host photoreceptors (Fig. 4D). We further investigated if graft RPE-strip can also reconstruct the basement membrane. In comparison to the intact site, the expression of type IV collagen was disrupted in the ablation area at 2 days after laser treatment (Fig. 4E) while hMERTK positive monolayered RPE cells engrafted in the RPE damaged area cells consistently showed basal expression of collagen type IV (Fig. 4F, G). Another example of histology from the M1 monkey eye is also shown in Supplemental Fig. 3. Transplanted hiPSC-RPE stably survived for 3 months, with its thickness decreasing over time in OCT images (Fig. S3A–C). HE staining revealed the presence of pigmented cells that were positive for the human surface marker STEM121 and hMERTK (Fig. S3D). These cells were also positive for RPE65 (Fig. S3E). For this eye, a 24G prototype cannula was used for transplantation, and an accumulation of inflammatory cells at the choroid beneath the graft was evident possibly due to the surgical invasion.

**Table 1:** Summary of hiPSC-RPE strip transplantation.

Monkey ID	Cell line	Number of RPE strips (RPE damaged area)	Number of RPE strips (normal area)	Observation period (months)	Canula	Comment
M1	201B7 modFucci	2 strips	None	1	24G	RHOK inhibitor^+^
M2	QHJI01	2 strips	2 strips	6	25G/31G	RHOK inhibitor^+^ Aseptic endophthalmitis
M3	QHJI01	2 strips	2 strips	6.5	25G/31G	RHOK inhibitor^−^
M4	QHJI01	2 strips	2 strips	3.5	25G/31G	RHOK inhibitor^+^

### hiPSC-RPE expanded to cover a larger area when transplanted in the RPE ablated area than in the intact area

**Figure 5:**
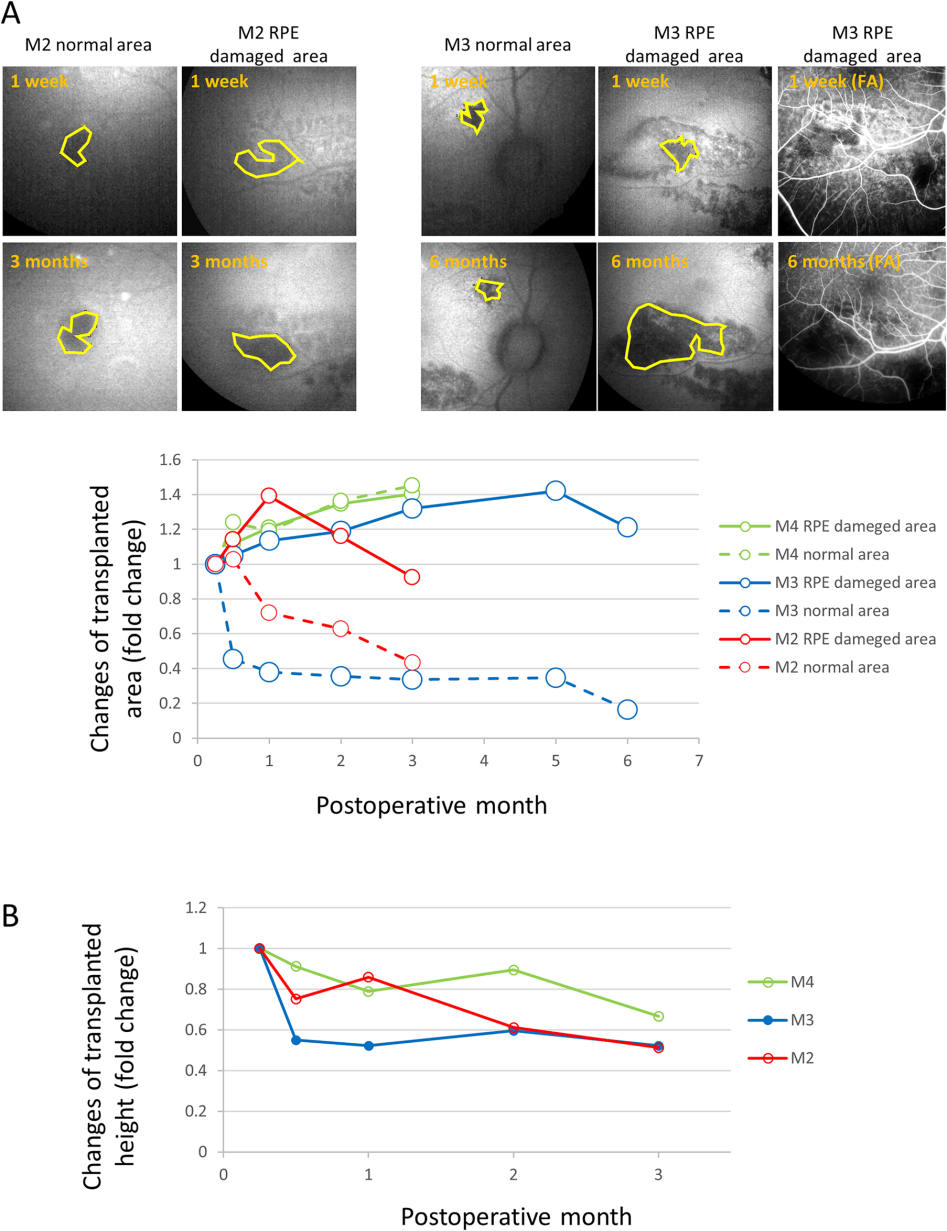
Temporal changes in engrafted area and graft thickness of hiPSC-RPE from baseline at 1 week after transplantation. (**A**) Changes in engrafted hiPSC-RPE. The graft area was calculated as a ratio to that at 1 week after transplantation as the baseline. The graft area was manually traced at the engrafted site which was well identified in the images of all time points. Examples of manual tracing using autofluorescence images are shown on top panels. FA images were also referred to determine the graft area as shown in the right top. (**B**) Changes in the thickness of the transplanted iPS-RPE. The thickness was calculated as a fold change than observed at 1 week after transplantation by the maximum thickness using the same sectional view of optical coherence tomography (OCT) images.

In three eyes with the optimized surgical protocol using a 31G cannula tip and an observation period of ≥ 3 months (M2–M4), the temporal changes in area and thickness of the transplanted grafts were quantitatively evaluated as a ratio to those at 1 week after transplantation (Fig. 5). In the RPE-damaged area, the graft coverage gradually expanded up to and over 3 months in two eyes, whereas the tendency was not observed in the eye that had aseptic endophthalmitis. In contrast, the engraftment area tended to decrease when hiPSC-RPE was placed at the RPE-intact site (Fig. 5A). This observation indicates that graft cells may not actively replace the healthy RPEs. The thickness of the grafts, regardless of whether they were in the normal or damaged area, rapidly decreased within 2 weeks postoperatively and gradually thinned until 3 months (Fig. 5B). This indicates that unnecessary cells would be swiftly removed in vivo. No incidence of tumorigenicity of the transplanted grafts was observed and very few cells were positive for proliferation marker Ki67 at the time of sacrifice (Fig. S4).

### Immune responses after hiPSC-RPE strip xenotransplantation

In this study, xenotransplantation (human RPE cells into monkey eyes) was performed by the systemic administration of cyclosporin. Although in vivo imaging showed that the graft survived for up to 6 months without showing any obvious signs of rejection, cell accumulation, probably inflammatory cells, was observed in the choroidal space at the graft site. To characterize the nature of this cell migration, we immunostained these cells using multiple immune cell markers (Fig. S5). Collectively, the cells beneath the graft RPE and in the choroid were strongly positive for CD3 (pan-T cell marker), Iba1 (microglia/macrophage marker), and CD4 (helper T cell marker), mildly positive for CD20 (B cell marker), IgG (antibody/B cell marker), and MHC2 (antigen-presenting cell/inflammatory cell marker), but negative for CD8 (cytotoxic cell marker) and NKG2A (natural-killer cell marker). This implies that the graft confronts the early phase of rejection. Although xenotransplantation can elicit a maximal immune response, graft immune rejection should be carefully monitored in clinical situations. Interestingly, even with this immune cell accumulation, transplanted RPEs were mostly observed as a monolayer in histological examinations on top of these accumulating cells.

## Discussion

In this study, we utilized a microsecond pulse laser to establish a primate RPE-damaged model. Immediately after the laser treatment, discontinuity and disturbance of the pigmented RPE cell layer were observed by HE staining and RPE65 immunostaining, suggesting moderate RPE ablation. The disruption of pigmented RPE cells was evident up to 2 days after laser treatment. However, on the fifth day, the remaining RPE cells and/or the healthy RPEs from the neighboring area started to migrate over the damaged area, so the transplantation was conducted 2 days after laser treatment. Overall, the photoreceptor cells appeared intact on HE and rhodopsin immunostaining, indicating the potential utility of this model for RPE-originated retinal diseases. The RPE injury site was also confirmed by dye leakage in FA that was conducted immediately after micropulse laser treatment. Histologically, laser treatment does not lead to complete ablation of RPE. However, our result showed that RPE damage or moderate ablation provides an environment for better expansion of RPE engraftment than those of the intact RPE sites. We previously reported that when hiPSC-RPE strips were placed in the ablated RPE area in a culture dish, the RPE expanded from the strip but halted its extension at the site of existing cells, forming ZO-1-positive junctions with the pre-existing RPEs, reflecting “contact inhibition”^10^. Consistently, the current results suggest that graft RPE cells do not actively replace the pre-existing RPEs, particularly the healthy ones. Since our microsecond pulsed laser treatment was able to cause a moderate RPE ablation with minimal damage to the photoreceptor cells and choroid, the protocol may be further optimized for clinical application to enhance RPE engraftment in RPE dysfunctional diseases in the future.

In the previous paper from our group by Ishida et al. reported the potential improvement in the engraftment of post-transplantation iPS-RPE cell suspensions by using the inhibitor of rho kinase (RHOK), which simultaneously suppressed apoptosis and enhanced cell adhesion^14^. While anticipating enhancements in adhesion and expansion effects of grafts in RPE strips, we could not obtain enough data to observe any significant change by the use of RHOK inhibitor in the current study.

Previously, Duarri et al. reported that cell adhesion of RPE cells is weak in areas of RPE atrophy with suspension transplantation^15^. RPEs may require some scaffold for engraftment, especially when transplanted as a cell suspension. In this study, monolayered graft RPE or the top layer of RPE aggregates showed the correct polarity with the expression of basal collagen type IV and apical MERTK, consistent with our previous report^10^. This observation indicates that once RPE strips are settled as cell aggregates, they may migrate out with collagen secretion and produce basement membrane, which may consistently support the engraftment of hiPSC-RPE strips to fill between the RPE gaps even in an unfavorable vivo environment with an insufficient scaffold. Further clinical studies should be performed to investigate whether the transplanted RPE cells could also expand over atrophic or diseased RPE cells. The correct polarity of grafted RPE, together with the host photoreceptor cells with outer segments over the graft area after 3 months of transplantation, was demonstrated in this study, supporting the functionality of these cells. In the current study, a partial decrease in ONL thickness may have occurred over the persisting bumpy graft clumps or due to immune-rejection by xeno-transplantation, and a careful monitoring of the overlying host ONL is required in clinical application.

In summary, sheet transplantation enables excellent visibility of the graft during surgical procedures, allowing for precise control over the delivery of the graft to the target site while preventing backflow. Furthermore, there is no concern about scaffolding for cell adhesion, even in areas with RPE atrophy. However, sheet transplantation requires a large incision for transplantation, which may not be appropriate for some cases like perimacular or diffuse/patchy RPE degeneration. In this study, we were able to perform transplantation of RPE strips through the standard 25G vitreous port using a 25/31G cannula, enabling multiple grafts through repeated insertions. This procedure may be utilized in the transplantation of RPE cells to perimacular or multiple patchy RPE atrophy areas. RPE strips did not exhibit cell backflow or diffusion, which was often observed in cell suspension transplantation. Additionally, apart from the insertion site, there was no apparent damage to the photoreceptor layer on the transplanted RPE. Collectively, hiPSC-RPE strip transplantation is considered a promising therapeutic approach for diseases involving RPE atrophy.

## Methods

### Animals

All animals were treated in accordance with the Association for Research in Vision and Ophthalmology statement for the use of animals in ophthalmic and vision research. Animal experiments were conducted with the approval of the Animal Research Committee at the RIKEN Center for Biosystems Dynamics Research. Six cynomolgus monkeys were obtained from Eve Bioscience Ltd. (Wakayama, Japan). All animals were housed under the standard 12 h light/dark cycle.

For laser photocoagulation, in vivo imaging, and hiPSC-retina transplantation, the monkeys were first anesthetized with intramuscular injections of ketamine hydrochloride (10 mg/kg) and xylazine (2 mg/kg), followed by periodic additional ketamine hydrochloride (5 mg/kg) and xylazine (1 mg/kg) for approximately every 30 min to maintain anesthesia when necessary. Pupils were dilated with 0.5% tropicamide and 0.5% phenylephrine hydrochloride, and topical 1% tetracaine or 0.4% oxybuprocaine hydrochloride was used for cornea anesthetization when needed.

### RPE ablation model

An IQ 577 MicroPulse Laser (Iridex) with a TxCell scanning laser delivery device was used to selectively damage the RPE using a Volk HR centralis contact lens (Volk Optical Inc.) with a 74° field of view, ×1.08 magnification, and laser spot magnification of ×0.93. Micropulse power sufficient to obtain RPE damage to the laser area (400–900 mW) and exposure times of 200 ms were used. The micropulse mode with duty cycles of 1% (0.05 ms pulse “on” and 4.90 ms pulse “off”) or 3% (0.05 ms pulse “on” and 1.60 ms pulse “off”) was used. A 0.25 mm^2^ RPE damage was created using a spot size of 100 µm and 5 × 5 confluent grids.

### Preparation of the hiPSC-RPE strip

#### Transplantation of hiPSC-RPE strips in monkey eyes

The hiPSC-RPE strips for transplantation were fabricated in 2 mm-width grooves of the culture device as described previously^10^. A total of 2.00 × 10^5^ cells were seeded in each groove and cultured for 2 days. The mean cell counts of the RPE strips at the time of transplantation was 1.59 ± 0.12 × 10^5^ (mean ± sd). The transplantation was planned 2 days after laser treatment in both the laser-conducted and intact areas (Fig. 3). For transplantation of hiPSC-RPE cells, a vitrectomy (Constellation^®^, Alcon, Geneva, Switzerland) was performed, followed by the detachment of the posterior vitreous membrane. Subsequently, the retinal detachment was gently made with Opti-MEM (ThermoFisher Scientific) with or without ROCK inhibitor Y-27632 (10 µM) using a 24G cannula or 38 g/25 g polytip cannula (MedOne 3219 PolyTip^®^ cannula 25 g/38 g) at the RPE-injured site first (Fig. 3, Table 1). The liquid drain hole was made on the other side of the retinal bleb with the same tip. The hiPSC-RPE strips were then gently inserted into the bleb with ×7 viscous medium (Viscoat 0.5, Alcon, Tokyo, Japan) using a 31/25 g Medone cannula. The injection was repeated when needed to achieve the transplantation of two strips. Perfluorocarbon (PFC, Alcon) was then placed to cover the insertion hole of the bleb, and the second retinal detachment was made on the upper side of the macula, and two RPE strips were inserted as previously described. Fluid-gas exchange was conducted, and the subretinal fluid was gently pushed out from the bleb under the gently tumbling PFC. The vitreous space was replaced with silicon oil after removing PFC.

### Post-operative in vivo examinations

The transplanted cells were monitored by color fundus photographs, FA (CX-1, Canon), and OCT (RS-3000; NIDEK) at 1, 2, 4, 8, and 12 weeks and 6 months after surgery. We transplanted hiPSC-RPE cells into monkey eyes with immunosuppressive medication with cyclosporin A (Sigma–Aldrich) at an initial dose of 20 mg/kg per day. We monitored the concentration of cyclosporin A in the serum of the monkey before and after surgery. In addition, a local steroid, triamcinolone acetonide, was administered to the subtenon (STTA, 20 mg/time), and the graft area (pixel/mm^2^) was measured using NIH Image J software after transplantation.

### Immunostaining procedures

The ocular tissue from the transplanted Cynomolgus monkey was preserved using SUPERFIX (Kurabo) and subsequently subjected to automated dehydration via the Excelsior system (ThermoFisher Scientific). This was followed by paraffin embedding using P3683 (Sigma–Aldrich) and sectioning to a thickness of 10 μm utilizing the AS-200 auto slide preparation system (Kurabo). For immunostaining, H&E sections were used to locate the graft cells and to detect the obvious symptoms of inflammatory immune responses. We then performed immunohistochemistry for RPE markers and rhodopsin and immune cells as previously described^10,16,17^. Sections were blocked with blocking one (Nacalai Tesque, Kyoto, Japan) or 5% goat serum in PBS for 1 h at room temperature and incubated with primary antibodies overnight at 4 °C. After rinsing with Tween 20 in PBS three times, the sections were incubated with secondary antibodies for 1 h at room temperature and counterstained with DAPI (×1000). For the co-immunostaining using two different mouse antibodies for Ret P1 and RPE65, the FlexAble CoraLite^®^488 antibody labeling kit for mouse IgG1 (Proteintech KFA021) was used according to the manufacturer’s instructions. We observed the samples, and images were obtained using Keyence BZ-X810, Zeiss LSM700, and Leica-TCS SP8 confocal microscopy platforms. Primary antibodies and their respective dilution ratios are listed in Supplementary Table S1.

### Quantitative evaluation of the graft coverage area

Analysis of the hiPSC-RPE transplanted area was performed with ImageJ according to the manufacturer’s instructions. After transplantation of hiPSC-RPE, we utilized autofluorescence images for the measurement of the engrafted area and also referred to FA images to confirm the engrafted area in the eyes that were followed up for ≥ 3 months. The graft hiPSC-RPE margin was manually marked, and the area was quantified. The optic disc area was then similarly quantified manually, and the engrafted area was normalized using the disc area on the same image. The engrafted area at 1 week post-transplantation was designated as a reference (value of 1), and the area ratios at 2 weeks and 1, 2, and 3 months post-transplantation were calculated. The engrafted area that could be well identified in the images of all the examined time points was chosen for quantitative assessment.

### Supplementary Information

Supplementary Information.

## Data Availability

The datasets generated and analysed during the current study are available from the corresponding author on reasonable request.
