# Supplementary material for: Graft cell expansion from hiPSC-RPE strip after transplantation in primate eyes with or without RPE damage
Source: Sci Rep. 2024 May 2;14:10044. doi: 10.1038/s41598-024-60895-w (PMC11065889; doi:10.1038/s41598-024-60895-w)
Supplement: Supplementary file 1 — Supplementary Information. [file 41598_2024_60895_MOESM1_ESM.pdf]

Supplementary Information

Graft cell expansion from hiPSC-RPE-strip after transplantation in primate eyes with or without RPE damage

Keisuke Kajita, Mitsuhiro Nishida, Yasuo Kurimoto, Satoshi Yokota, Sunao Sugita, Toshika Senba, Satoshi Shirae, Naoko Hayashi, Atsushi Ozaki, Yoko Miura, Akiko Maeda, Yoshinori Mitamura, Masayo Takahashi, Michiko Mandai

<u>Supplemental Figures</u>

Figure S1: Images of hiPSC-RPE immediately after hiPS-RPE strip transplantation (M4)

Figure S2: Temporal change of hiPS-RPE-Strips after transplantation at the RPE damaged Site (M1)

Figure S3: Specific human MERTK positivity on hiPSC-RPE

Figure S4: Figure S5: Proliferative cells in hiPSC-RPE strips after transplantaion

Figure S5: Immune responses after hiPS-RPE strip xenotransplantation

<u>Supplemental Table</u>

List of antibodies

<u>Supplemental methods</u>

<u>Supplemental video</u>

Video-S1 loading and release of the hiPS-RPE strip using a 25/31G cannula.

A

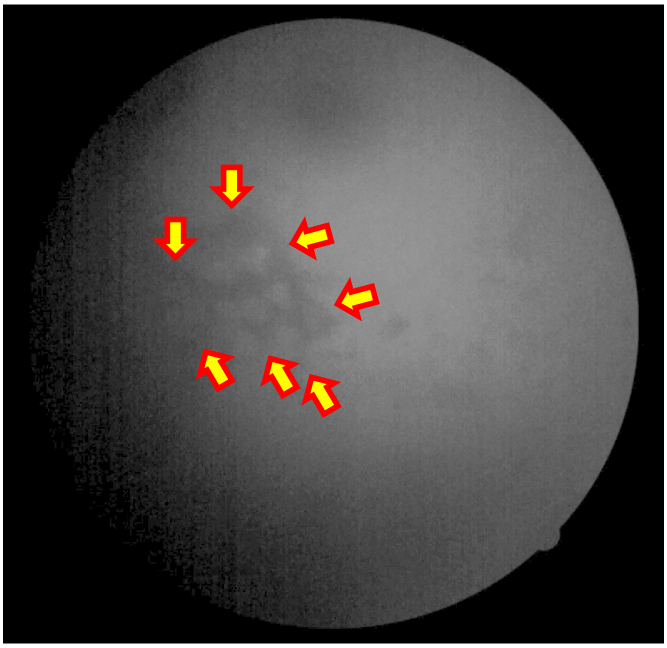

B

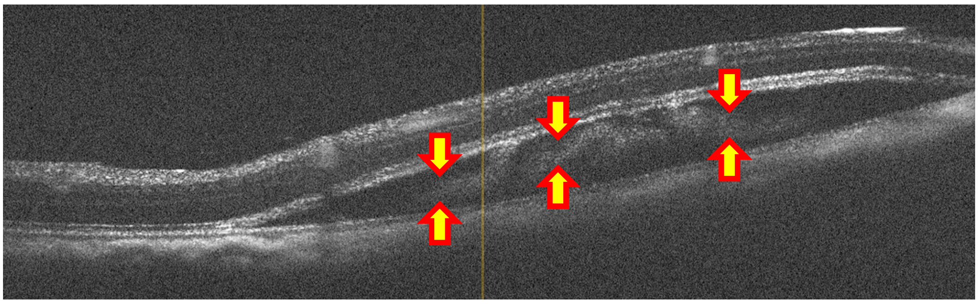

Figure S1: hiPSC-RPE strips immediately after hiPS-RPE strip transplantation (M4)

A. The autofluorescence image captured immediately after the transplantation of RPE strips. The strip figure of transplanted RPE was observed as low fluorescent figure.
B. An optical coherent tomography (OCT) image taken immediately after the transplantation of RPE strips, revealing the transplanted RPE as cord-like hyper reflectivity observed beneath the retina.

A

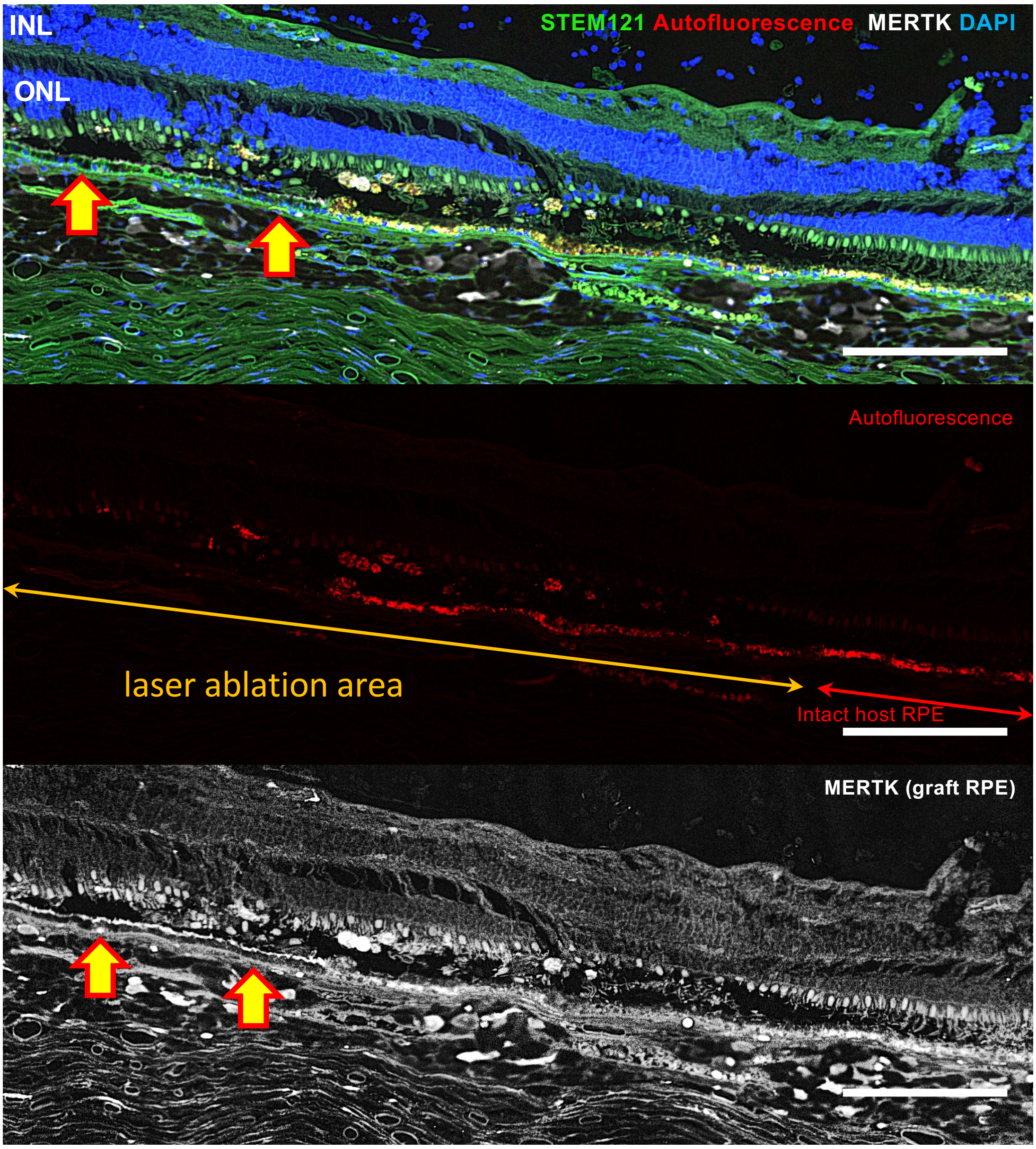
INL
ONL
STEM121 Autofluorescence MERTK DAPI
Autofluorescence
laser ablation area
Intact host RPE
MERTK (graft RPE)

B

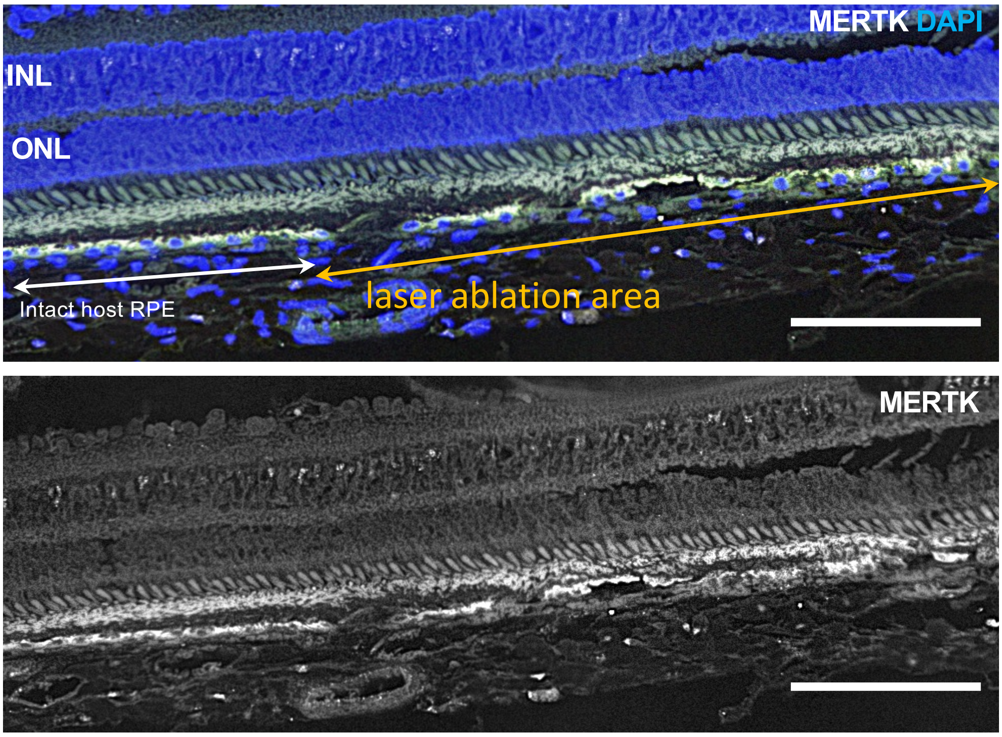
MERTK DAPI
INL
ONL
Intact host RPE
laser ablation area
MERTK

Figure S2: Specific anti-MERTK positivity pattern on hiPSC-RPE cells

A. The transplanted RPE on the laser ablation site express STEM121 on cell membrane and MERTK apically (arrows), which are both free of autofluorescence. Fuzzy staining in the laser ablation site and in the intact host RPE by the MERTK antibody do not show the specific apical pattern and overlaps with the autofluorecence (channel 2 with no antibody).
B. No specific pattern of human MERTK positive signal was observed in the areas of intact and laser treated RPEs

ONL, outer nuclear layer; INL, inner nuclear layer

Scale bars: (A), 300 µm; (B), 200 µm

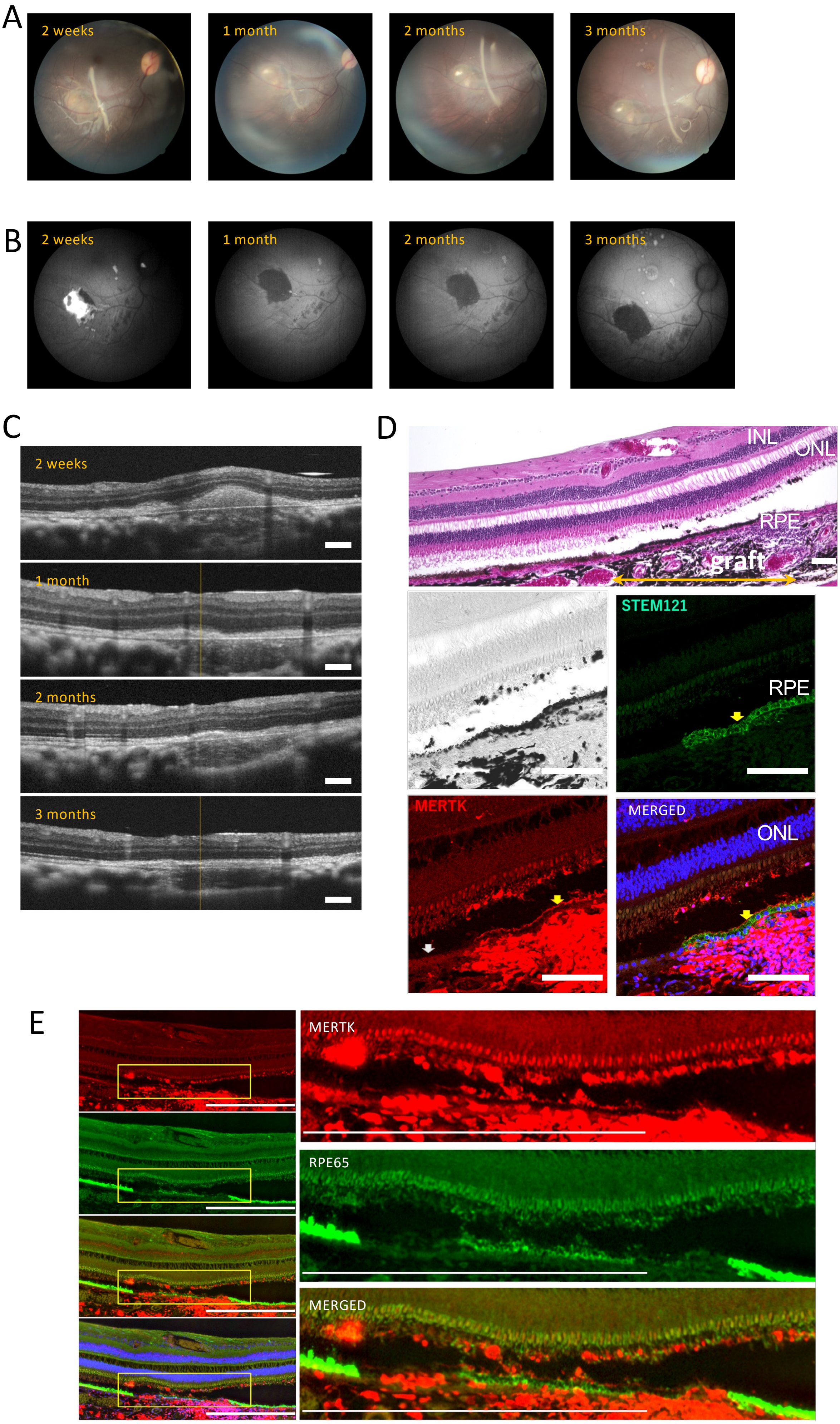

A
2 weeks
1 month
2 months
3 months
B
2 weeks
1 month
2 months
3 months
C
2 weeks
1 month
2 months
3 months
D
INL
ONL
RPE
graft
STEM121
RPE
MERTK
MERGED
ONL
E
MERTK
RPE65
MERGED

Figure S3: Temporal change of hiPS-RPE-Strips after transplantation at the RPE damaged Site (M1)

A. Temporal changes of hiPS-RPE-strips on color fundus images.
B. Autofluorescence image of hiPS-RPE strips after transplantation. hiPS-RPE graft showed strong fluorescence at 2 weeks due to the expression of mCherry by the non-dividing cells in M8 modFucci line. Fluorescence became weak by increased graft pigmentation at 1 month and later after transplantation.
C. OCT images of hiPS-RPE strips after transplantation. The height of graft cells were decreased over time to become almost flat at 3 months.
D. hiPS-RPE strips in HE staining. These cells express human marker STEM121 on cell membrane and phagocytosis marker MERTK on the apical surface (yellow arrow), while monkey RPEs in the intact area on the left side show no such specific staining pattern with the current MERTK antibody (white arrow).

E. Transplanted RPE cells express RPE 65 and MERTK. This eye also had marked accumulation of macrophages in the choroid beneath the xeno-graft, which are also strongly positive for MERTK.

ONL, outer nuclear layer

Scale bars: (C), 300 µm; (D), 100µm; (E), 500µm

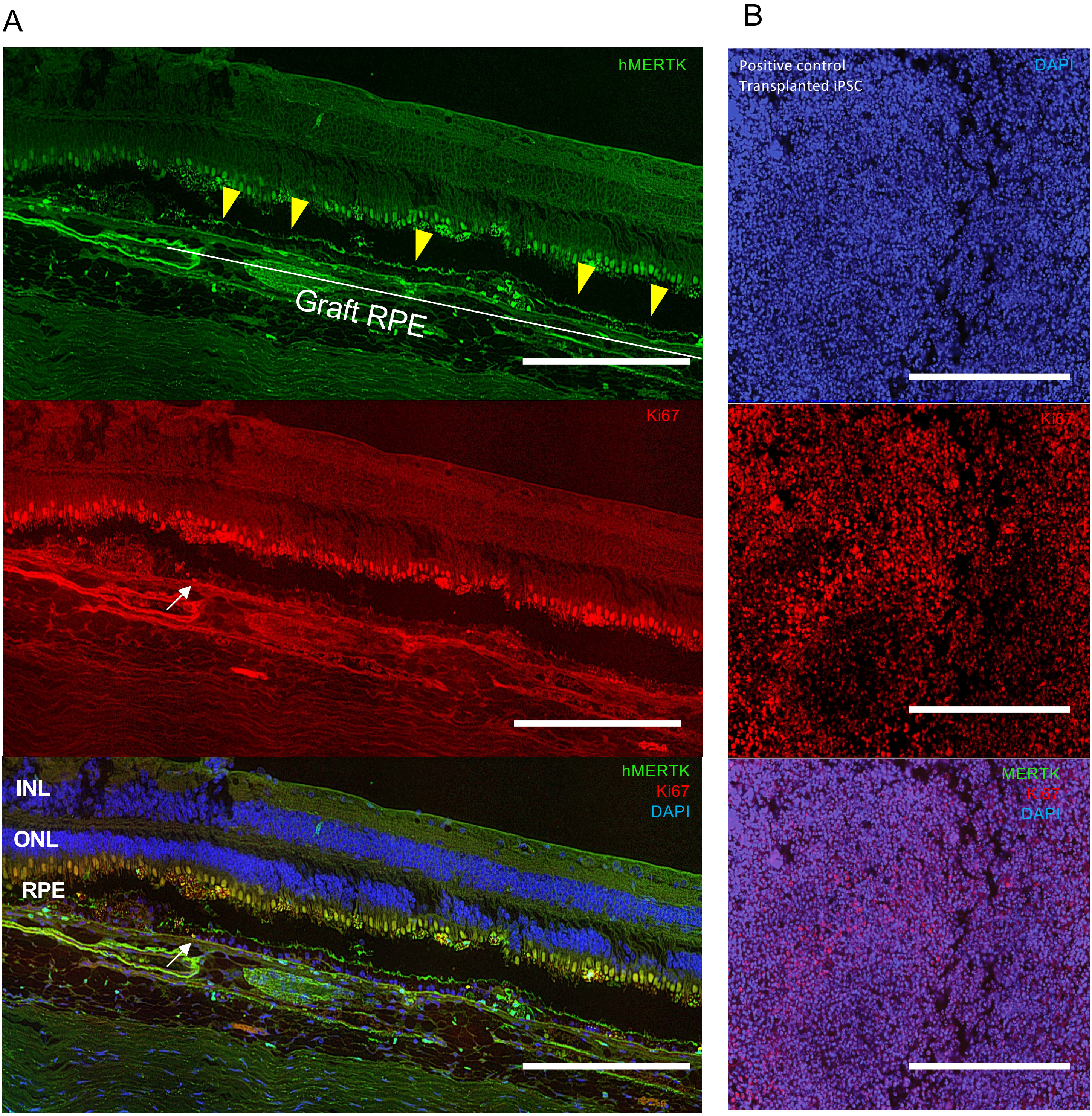

Figure S4: Proliferative cells in hiPSC-RPE strips after transplantation

A. Proliferative marker Ki67 was positive in very few cells within hiPSC-RPE graft at the time of sacrifice. Typical apical immunostaining pattern of hMERTK (yellow arrowheads) indicates the presence of grafted RPE cells in the white bar area (top panel).
B. Positive control staining; proliferating human iPSCs were transplanted in a nude rat eye and processed with the same paraffin section/immunostaining procedure.
ONL, outer nuclear layer; INL, inner nuclear layer
Scale bars: 300 µm

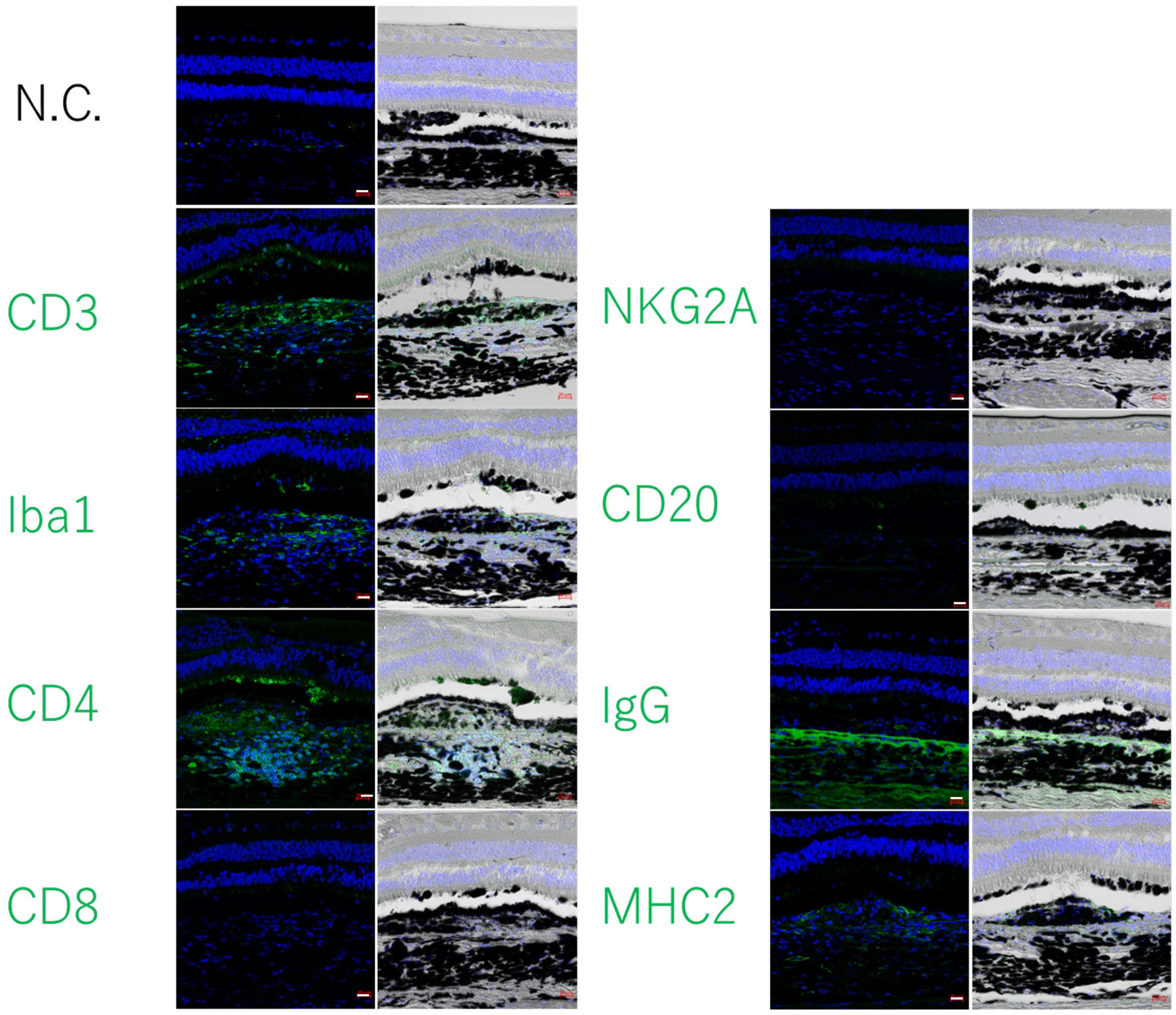

Figure S5: Immune responses after hiPS-RPE strip xenotransplantation

The grafted RPE were mostly observed as a monolayer, but there was an accumulation of immune cells at the graft site, which were strongly positive for CD3, Iba1,CD4 and mildly positive for CD20, IgG, and MHC2 but negative for CD8 and NK. This indicates that the graft confronts with the early phase for rejection.

Scale bars 20um

Supplemental Table 1 List of antibodies

Antibody	Host	Manufacture	catNo.	Dilution
RPE65	mouse	Milipore	MAB5428	1:300
MERTK	rabbit	abcam	ab52968	1:200
Collagen type4	mouse	abcam	Ab6311	1:400
Stem121	mouse	Takara	Y40410	1:200
RetP1(RHO)	mouse	sigma	O4886	1:500
Ki67	mouse	BD	550609	1:200
CD3	rabbit	abcam	ab16669	1:100
CD4	rabbit	abcam	ab133616	1:100
CD8 alpha	rabbit	abcam	ab4055	1:500
CD8 alpha	mouse	abcam	ab17147	1:200
CD20	rabbit	abcam	ab78237	1:100
Iba1	rabbit	Wako	019-19741	1:1000
Human IgG	rabbit	abcam	ab109489	1:300
Human HLA-DP, DQ, DR Antigen	mouse	Dako	M0775	1:100
NKG2A	rabbit	abcam	ab93169	1:100
